# Monitoring Cannabinoids and the Safety of the Trace Element Profile of Light *Cannabis sativa* L. from Different Varieties and Geographical Origin

**DOI:** 10.3390/toxics10120758

**Published:** 2022-12-05

**Authors:** Vincenzo Nava, Ambrogina Albergamo, Giovanni Bartolomeo, Rossana Rando, Federica Litrenta, Giovanna Lo Vecchio, Mario Concetto Giorgianni, Nicola Cicero

**Affiliations:** 1 Department of Biomedical, Dental, Morphological and Functional Image Sciences (BIOMORF), University of Messina, 98100 Messina, Italy; 2 Science4Life Srl, an Academic Spin-Off of University of Messina, Viale Annunziata, 98100 Messina, Italy; 3 Azienda Ospedaliera Universitaria Policlino “G. Martino”, Viale Gazzi, 98100 Messina, Italy

**Keywords:** light cannabis, hemp, THC, CBD, CBN, potentially toxic elements, consumer safety, botanical variety, geographical origin

## Abstract

Non-intoxicating *Cannabis sativa* L. (i.e., hemp) is increasingly used for industrial, cosmetic and food applications. Despite the fact that the EU has not yet established a regulatory framework on hazardous compounds (i.e., toxic inorganic elements), the monitoring of cannabis products is of paramount importance to safeguard consumers, also in view of the species propensity to accumulate toxic metals from the environment. The aim of this work was to assess the compliance with Law n. 242/2016 and the safety of hemp inflorescences from different varieties and Italian regions by monitoring the main cannabinoids and trace elements. All samples complied with the Italian law due to a THC content <0.6% counterbalanced by high CBD levels. However, inflorescences grown in Apulia and Lombardy, and from Finola and Tiborzallasi varieties accumulated higher Cu, Co, Cr, Ni and Pb contents than products from other producing areas (i.e., Sicily and Latium) and varieties (i.e., Antal, Futura75, Kompolti and Carmagnola), being moreover above the limits set by the US Pharmacopeia. A principal component analysis confirmed that trace elements, including toxic metals, were mainly affected by the geographical origin more than the botanical variety. Overall, this study highlights the need to continuously monitor hemp products and establish the earliest related regulatory frameworks.

## 1. Introduction

The term “cannabis” refers to the plant native to Central Asia which belongs to the genus of Cannabaceae of the Moraceae family. Its taxonomy has always been quite complex and troublesome. However, according to the most accepted interpretation proposed by Small and Cronquist (1976) [1], the genus is monospecific (Cannabis sativa L.), and includes two subspecies (Cannabis sativa L. subsp. sativa and Cannabis sativa L. subsp. indica (Lam.)) which are, in turn, characterized by several varieties, conferring to the genus a relevant genetic variability [2,3]. Cannabis varieties show a variable degree of adaptation to different environmental conditions, versatility and suitability for processing, also in relation to the geopedoclimatic conditions of growth and agronomic requirements [4,5]. *C. sativa* L. is a herbaceous plant with an annual cycle, and it is a dioecious species [5] with female or male flowers borne either individually or in conspicuous inflorescences. However, only the female inflorescences can produce more than 100 cannabinoids, the most important ones being Δ^9^- tetrahydrocannabinol (THC), cannabidiol (CBD) and cannabinol (CBN) [6]. THC is well known to induce psychoactive effects by directly acting on the cannabinoid receptors mostly present in the brain areas devoted to temporal and sensory perception, pleasure, thinking, memory and coordination [7,8]; while CBD and CBN are molecules with no psychotropic effects and different therapeutic properties, such as pain-relieving and relaxing effects, as well as antibacterial, antioxidant and anti-inflammatory activities [6]. Additionally, CBD may contrast some negative effects of THC [9]. The THC and CBD content, and the derived THC/CBD ratio, notoriously allow it to establish the type of cannabis, or its “chemotype” [5]. On this basis, three categories can be outlined: (i) the cannabis with chemotype >1 (chemotype I), THC between 0.3–20% and CBD <0.5%, which is typically exploited for the preparation of well-known drugs, such as hashish and marijuana; (ii) the cannabis with chemotype = 1 (chemotype II), THC between 0.3–2% and CBD >0.5%, which can be employed for the treatment of various pathological conditions, such as chronic pain, multiple sclerosis or spinal cord injury, as well as anxiety or depressive disorders [10]; and (iii) the cannabis with chemotype <1 (chemotype III), THC <0.3% and CBD, typically varying between 2–3%, and potentially reaching up to 40%, is commonly defined as “industrial” or “light” cannabis or, more simply, hemp [11]. According to the Council Directive 2002/53/EC, the cultivation of hemp is allowed by using seeds only from certain certified cannabis varieties, which are included in the EU catalog updated from year to year [12]. EU Regulations no. 1307/2013 and 1155/2017 and the Italian Law n. 242/2016 encourage the cultivation of such cannabis to produce food, cosmetics and semi-finished products (i.e., fiber, powder, oil or fuel), as well as material aimed at bioengineering, green building or phyto-remediation of contaminated sites [13,14]. Lately, such products have been also defined by the European Monitoring Centre for Drugs and Drug Addiction (EMCDDA) as “Low-THC cannabis products” [15].

Overall, hemp has been achieving an incredible success, and an unprecedented consumer demand for its non-intoxicating products has been observed, generating, however, the urgent need for further fundamental research, and new legislative, regulatory and business frameworks as well [16,17].

Many studies have already defined hemp as an excellent phytoremediation agent, since it can adsorb from soil and accumulate potentially toxic elements, such as lead (Pb), nickel (Ni), cadmium (Cd), arsenic (As), mercury (Hg) and chromium (Cr), thanks to the type of root it possesses [18,19,20]. Hemp has already been tested for its heavy metal tolerance and accumulation in semi-natural conditions [20], and for cleaning up environments with emulated Chernobyl conditions based on radiocesium levels (^137^Cs). The latter experiment, specifically, led to the detection of radioactivity in all plant tissues, as well as retting water, fiber and seed oil that could potentially end up in the hands of consumers [21]. However, there are several factors that may influence the extent of adsorption and accumulation of metals by *C. sativa* L. These include soil pH, its organic matter content, redox potential, clay content, cation exchange capacity, nutrient balance, humidity and temperature [4]. 

While hemp has proved to be valuable for the soil remediation of contaminated areas, its tendency to adsorb toxic elements from soil may pose a serious threat to consumers [21]. This is especially true for the extracts from the hemp inflorescence and fruit, which are considered “Novel Food” due to the relevant levels of CBD and other non-psychoactive cannabinoids, according to the Regulation (EU) n. 2283/2015. Nonetheless, the law calls also for the continuous evaluation of the safety of any product able to be placed on the EU market as novel food or food ingredient [22]. For this reason, the levels of metals present in hemp products should be constantly monitored, and suitable regulatory standards should be developed to keep them under control. 

On this basis, an array of inflorescences produced in four Italian areas and belonging to different varieties of *C. sativa* L. (chemotype III) were investigated to evaluate the compliance with the Law n. 242/2016, by monitoring the contents of main cannabinoids, and their safety, by the screening of trace elements. The experimental dataset was statistically explored to evaluate the power of cannabinoids and trace elements on the discrimination of investigated inflorescences based on botanical variety and/or geographical origin.

## 2. Materials and Methods

### 2.1. Samples

In this study, 24 types of dried inflorescences from Cannabis sativa L. (chemotype III) were provided during April–June 2022 by seven agricultural cooperatives distributed throughout Italy and dedicated to the outdoor cultivation of industrial hemp and the consequent processing of the inflorescences. For the sampling, six different varieties of *C. sativa* L. (i.e., Finola, Antal, Futura 75, Tiborzallasi, Kompolti and Carmagnola) grown in four Italian regions (i.e., Sicily, Lazio, Apulia and Lombardy) were collected in triplicate, for a total of 72 samples. Further details on the inflorescences, their variety and geographical origin are shown in Table 1. All inflorescence samples were packed in polyethylene bags and transported to the laboratory, where they were removed from their twigs, the ground, and passed through a 150-mesh sieve to obtain fine powders. All samples were stored at room temperature until further analysis. 

### 2.2. Material and Reagents

#### 2.2.1. ICP-MS Analysis

Nitric acid (HNO_3_, 65% *v/v*) and hydrogen peroxide (H_2_O_2_, 30% *v/v*) were of Suprapure grade and purchased from J. T. Baker (Mallinckrodt Baker, Milan, Italy). Ultrapure water (<5 mg/L Total Organic Carbon (TOC) was supplied by Merck-Millipore (Darmstadt, Germany). The commercial standard solution of Re (internal standard), and the standards of Al, Cr, Mn, Fe, Co, Ni, Cu, Zn, As, Se, Mo, Cd and Pb used for the calibration curves came from Supelco (Bellefonte, PA, USA). For the element analysis, all the equipment for sample collection, handling and storage, as well as laboratory glassware and polytetrafluoroethylene (PTFE) digestion vessels, were washed with 5% HNO_3_ before use. 

#### 2.2.2. GC-FID Analysis

Organic solvents, such as petroleum ether and chloroform, were ACS reagent grade and were provided by Merck (Darmstadt, Germany). THC, CBD and 4-androstene-3,17-dione (internal standard) were from Supelco (Bellefonte, PA, USA). 

#### 2.2.3. DMA-80 Analysis

A stock standard solution of Hg (Hg(NO_3_)^2^ in 10% HNO_3_, 2 mol/L, 1000 mg/L) was available from Merck-Millipore (Darmstadt, Germany). 

### 2.3. Cannabinoid Analysis 

Around 0.2 g of sample was weighed, mixed with 2 mL of petroleum ether, and sonicated in an ultrasonic bath (FALC instruments ultrasonic, Treviglio, Italy) for 15 min at room temperature. Subsequently, the sample was filtered and evaporated by means of a rotary evaporator (Buchi B-491, Büchi, Flawil, Switzerland). Hence, the dried extract was resuspended in chloroform, added with 0.5 mL of the internal standard 4-androstene-3,17-dione (final concentration, 3mg/mL) and injected into a gas chromatograph (GC) equipped with a split/splitless injector and a flame ionization detector (FID) (Dani Master GC1000, Dani Instrument, Milan, Italy). A SPB-1 column (15 m × 0.2 mm i.d., 0.2 μm film thickness, Supelco, Sigma Aldrich, St. Louis, MO, USA) was used. 

For the analysis, the following operating conditions were employed: column oven temperature from 200 °C (hold time 5 min) to 280 °C (hold time 17 min) at 4 °C/min; injector and detector temperatures, 280 °C. Carrier and makeup gases were He: 30 cm/s, N_2_: 25 mL/min; H_2_: 40 mL/min, air: 280 mL/min. The injection volume was 1 μL, with a split ratio of 1:100 and a split flow of 50 mL/min. Data handling and acquisition were performed by means of the Clarity Chromatography Software v. 4.0.2 (Dani Instrument, Milan, Italy). Cannabinoids such as THC, CBD and CBN were identified by direct comparison with the retention times of reference compounds and quantified by appropriate external calibration curves with an internal standard normalization. All samples were run in triplicate. 

### 2.4. Element Analysis

Element analysis involved a sample pretreatment based on a process of acid digestion conducted in a closed-vessel microwave digestion system (ETHOS 1, Milestone, Bergamo, Italy). Approximately 0.3 g of each sample was accurately weighed in PTFE vessels, added with 1 mL of internal standard Re at a concentration of 0.5 mg/L, and mixed with 7 mL of HNO_3_ and 1 mL of H_2_O_2_. The operating conditions of the digestion program were: 20 min at a temperature ranging from 0 to 180 °C (step 1), 15 min at a constant temperature of 180 °C (step 2) with a constant power microwave of 1100 W. Subsequently, samples were cooled at room temperature, diluted to a volume of 25 mL with ultrapure water and filtered using 0.45 μm PTFE filters. Both the blank solution (HNO_3_ and H_2_O_2_, 7:1 v/v) and the certified reference material (white clover- BCR402, Institute for Reference Materials and Measurements, European Commission, Joint Research Centre, Belgium) were prepared under the same conditions as the samples. The certified reference material (white clover- BCR402) contained the following mineral elements: Cr, Fe, Co, Ni, Zn, As, Se, Mo.

The screening of trace essential (Fe, Mn, Cu, Mo, Zn, Se, Co, Cr, Ni) and potentially toxic elements (As, Cd, Pb, Hg, Al) was carried out by the single quadrupole inductively coupled plasma-mass spectrometer (ICP-MS, iCAP-Q, Thermo Scientific, Waltham, USA) powered by a 27 MHz radiofrequency solid-state generator, and equipped with a PFA cyclonic spray chamber with a port accepting a 4 mm i.d. and 6 mm o.d. nebulizer, nickel sampler and skimmer cones of 1.1 mm and 0.5 mm. The instrument was also provided with an autosampler (ASX520, Cetac Technologies Inc., Omaha, NE, USA) coupled to an integrated sample introduction system. 

Before analysis, the ICP-MS method was optimized to reduce spectral (polyatomic and isobaric) and non-spectral interferences potentially affecting the multianalyte determination. For each investigated analyte, isobaric interferences were monitored by means of its several isotopes and corrected according to the most abundant isotope. As a result, ^27^Al,^52^Cr, ^55^Mn, ^56^Fe, ^59^Co, ^60^Ni, ^63^Cu, ^66^Zn, ^75^As, ^80^Se, ^98^Mo,^114^Cd and ^208^Pb were the isotopes monitored in all samples. On the other side, non-spectral interferences were reduced by means of online internal standards with a mass number close to that of the analytes to minimize instrumental drifts and matrix effect. Hence, ^45^Sc was chosen to monitor the signal of ^52^Cr, ^55^Mn, ^56^Fe and ^59^Co; while ^73^Ge for ^60^Ni, ^63^Cu, ^66^Zn, ^75^As, ^80^Se and ^98^Mo; ^115^In for ^111^Cd; and ^209^Bi for ^208^Pb. 

Inflorescence samples were then analyzed according to the following conditions: RF power, 1550 W; plasma gas (Ar) flow rate, 14 L/min; auxiliary gas (Ar), flow rate, 0.8 L/min; carrier gas (Ar) flow rate, 1.1 L/min; collision gas (He) flow rate, 4.7 mL/min; spray chamber temperature, 2.7 °C; sample depth and sample introduction flow rate, respectively 5 mm 0.93 mL/min. Integration times were 0.5 s/point for V, Fe, Se and As, and 0.1 s/point for the other elements. To integrate the peaks, 3 points for each mass and 3 replicate acquisitions were taken. Thermo Scientific Qtegra™ Intelligent Scientific Data System software (Thermo Scientific, Waltham, MA, USA) was employed for instrumental control and data acquisition. An external calibration procedure, based on the construction of seven-point calibration plots, with an internal standard normalization was adopted for quantitative purposes. All samples were analyzed in triplicate, along with analytical blanks. 

Hemp samples were also assessed for the Hg content by a direct mercury analyzer (DMA-80, Milestone S.r.l., Italy), based on the thermal decomposition amalgamation-atomic absorption spectrophotometry (TDA-AAS). DMA-80 was used instead of ICP-MS for several reasons. Firstly, it is a more versatile analytical instrumentation than ICP-MS, as it allows direct sample analysis, without the need for pre-treatment. In addition, operators do not encounter mercury exposure. Another important reason is that mercury analysis by ICP-MS requires the use of additional instrumental devices to clean the entire instrumentation, as mercury tends to accumulate in different parts and cause saturation phenomena in the instrument. This problem is overcome, however, in the DMA-80 where mercury vapors are trapped thanks to a gold amalgamator inside the instrumentation. 

Specifically, according to the EPA method 7473 (SW-846) [23]. Briefly, ~100 mg of every sample was initially dried at 200 °C for 3 min and subsequently thermally decomposed at 650 °C for 2 min. The Hg content was then determined by measuring absorbance at 253.7 nm according to an external calibration, exploiting a seven-point calibration curve. 

### 2.5. Validation of the ICP-MS and DMA-80 Methods

Linearity, the limit of detection (LOD), limit of quantification (LOQ), accuracy and precision were validated for every analyte investigated, following the criteria established by Eurachem [24]. Data from the validation process are reported in Table 2. 

The linearity of the signal concentration for every analyte was determined based on the linear least-square regression method. To this purpose, multi-standard solutions were used to construct seven-point calibration curves in the range 0.5–50.0 μg/L. However, the concentration range of the Hg curve was 5–100 μg/L. The obtained R^2^ values ranged from 0.9992 (for Cu) to 0.9999 (for Cd). The following experimental formulas were used to calculate LOD and LOQ: 3.3 σ/b and 10 σ/b, respectively, where σ is the standard deviation of the analytical blank (*n* = 6) and b is the slope of the relative calibration curve. LODs ranged, respectively, from 0.001 μg/kg and 0.003 μg/kg (Co, As and Cd) to 0.067 μg/kg and 0.221 μg/kg (for Al) (Table 2).

For the accuracy assessment, the BCR-402 (white clover) was analyzed in six replicates, and the difference between the mean experimental value and the reference value was reported as mean percent recovery. However, the accuracy of elements non-present in the reference material was estimated by the surrogate recovery [25]. In a separate experiment, a sample of inflorescences was spiked with a known amount of elements such as Al, Mn and Cu and analyzed in replicate alongside the same unspiked sample. The mean difference between these two values corresponded to the recovered part of the spiked analyte. The lowest and the highest average recovery were observed, respectively, for Cu (96.00%) and Fe (102.50%). Repeatability was evaluated in terms of precision, by considering the analyses of the certified matrix and the spiked sample performed in the same day, and intermediate precision, by considering a longer period of time (1 week). For the evaluation of precision, six replicates were carried out. Precision and intermediate precision, expressed as relative standard deviation (RSD%), were, respectively, below 1.3% and 1.7% (Table 2). 

### 2.6. Statistical Analysis

The statistical data elaboration was performed by R studio version 3.6.1 (R Studio: Integrated Development for R., Boston, MA, USA) for Windows. A descriptive analysis, including mean, median and standard deviation, of inorganic elements and cannabinoids overall inflorescence samples was conducted and shown in Tables S1 and S2. After running a Shapiro–Wilk test to verify the normal distribution of experimental data, the one way-ANOVA was applied for every independent variable to produce an F-statistic, i.e., the ratio of the variance calculated among the means to the variance within the samples. Statistical significance was accepted when Fcalculated > Fcritical (Fcritical = 1.75 with α < 0.001). 

Then, an exploratory PCA was performed to explore sample discrimination in relation to the botanical variety and/or geographical origin of inflorescences, as already discussed elsewhere [26,27,28]. 

## 3. Results and Discussion

### 3.1. Cannabinoids

**Figure 1:**
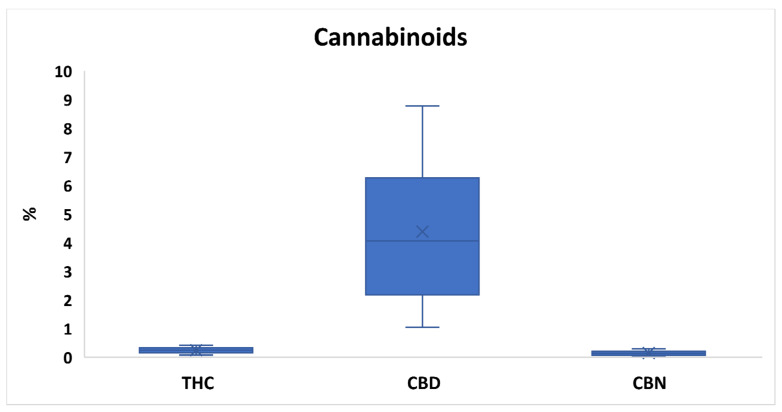
Box plot reporting the variable percentage of cannabinoids (Δ^9^- tetrahydrocannabinol (THC), cannabidiol (CBD) and cannabinol (CBN)) in investigated hemps. The “×” indicates the average value of each cannabinoid.

Figure 1 and Table S1 (Supplementary Material) report the variable percentage contents of the main cannabinoids (i.e., THC, CBD and CBN), determined in hemp samples by GC-FID, with results expressed on a dry weight basis. Based on the one-way ANOVA, the Fcalculated of each cannabinoid is also shown in comparison with the set Fcritical. Although the F values of THC, CBD and CBN were generally much lower than those identified for the elements, they were still higher than the Fcritical value (11.35–45.30 vs. 1.75), as they significantly varied between investigated samples (Table S1). 

All hemp samples complied with the Council Directive 2002/53/EC on the common catalogue of varieties of agricultural plant species and Italian Law 242/2016 on the provisions for the promotion of hemp cultivation and its agro-industrial supply chain, due to a percentage content of the active ingredient THC <0.6%. The THC ranged from 0.08% to 0.42%, with mean and median levels, respectively, equal to 0.24% and 0.25%. The consistency between mean and median THC content highlighted a little variation of such cannabinoid over hemp samples. Coherently, THC showed the lowest F value (11.35). When determining the THC content of Cannabis products by GC-FID, the effect of THCA decarboxylation to THC due to the high instrumental temperature of the injector should be considered. In our case, however, a minimal and negligible effect of decarboxylation may be assumed, as the THC obtained in all hemps still did not exceed the legal 0.6%. With reference to the geographical origin, products from Latium and Lombardy showed in general the highest and lowest THC levels (0.10–0.30% and 0.15–0.42%, respectively). Concerning the variety, Tiborzallasi hemps and Carmagnola inflorescences were characterized by the most and least abundant levels of the psychoactive molecule (0.10–0.37% and 0.08–0.20%, respectively) (Table S1). 

In light hemp, the lower levels of THC are typically juxtaposed with the higher levels of CBD, a cannabinoid with anti-seizure activity, also, it has anti-inflammatory, anti-tumor, analgesic and anti-psychotic activity [29]. In the present study, CBD ranged from 1.05% to 8.78%, with mean and median contents of 4.39% and 4.06%. Overall, a consistent variation of such cannabinoids over Italian hemps was pointed out, as confirmed also by the higher F value of 45.30. Apulian and Latium hemps showed the most and least abundant CBD content (5.44–8.78% and 1.05–3.26). On the other hand, Kompolti and Finola varieties reported in general the highest and lowest CBD levels (1.74–8.78% and 2.92–5.74%) (Table S1). The cannabinoid CBN is known for its sedative, anti-inflammatory and anti-microbial effects [29]. Such a molecule has also been experimentally demonstrated to stimulate the appetite as an efficient and non-toxic alternative to THC [30]. According to Table S1, CBN varied between 0.03–0.30% with mean and median values, respectively of 0.14% and 0.13%. The F value of 18.01 indicated a lower variation of such cannabinoid over-investigated hemps than CBD. In relation to the geographical origin, products from Sicily and Latium were the least and most concentrated in CBN (respectively, 0.04–0.12% and 0.11–0.27%). On the other hand, according to the variety, Finola and Tiborzallasi inflorescences showed the highest and lowest content of such cannabinoid (respectively, 0.03–0.14% and 0.11–0.30%). 

### 3.2. Element Profile

**Figure 2:**
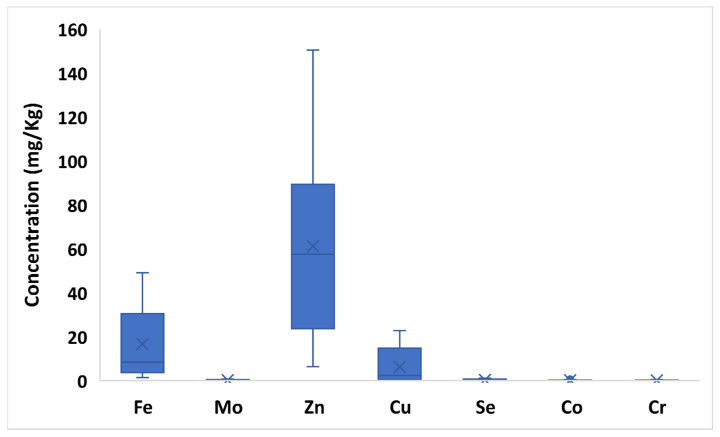
Box plot of essential trace elements (Fe, Mo, Zn, Cu, Se, Co, Cr) concentration in hemps. The “×” indicates the average value of each element.

**Figure 3:**
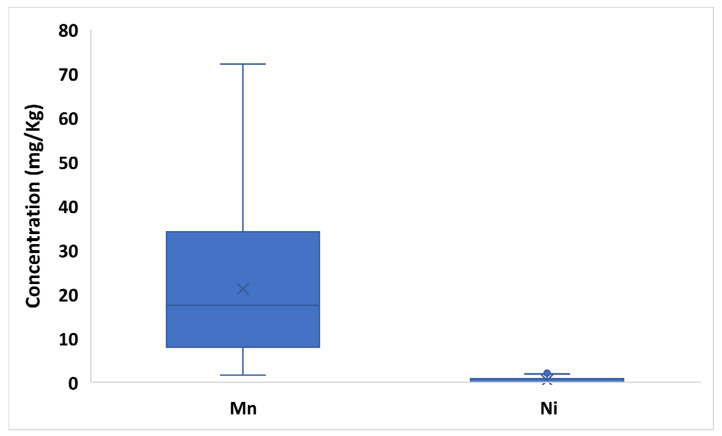
Box plot of probably essential trace elements (Mn, Ni) concentration in hemps. The “×” indicates the average value of each element, whereas the outlier points display the outlier data lying either below the lower whisker line or above the upper whisker line.

**Figure 4:**
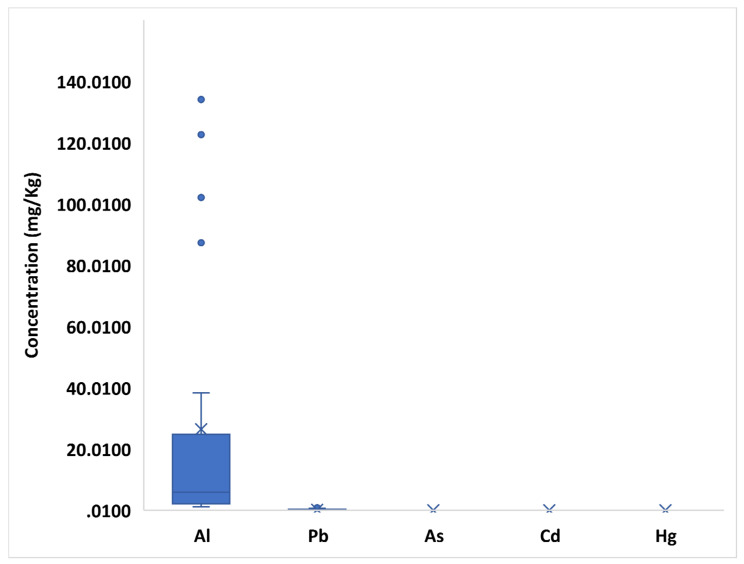
Box plot of potentially toxic element (Al, Pb, As, Cd, Hg) concentration in hemps. The “×” indicates the average value of each element, whereas the outlier points display the outlier data lying either below the lower whisker line or above the upper whisker line.

Although various (and controversial) classifications of trace elements have been proposed, elements with nutritional significance from this study will be classified into essential elements, probably essential elements, and potentially toxic elements according to WHO and subsequent updates [31,32]. Table S2 (Supplementary material) and Figure 2, Figure 3 and Figure 4 show the variability of the trace element profile over the hemp samples revealed by validated ICP-MS and TDA-AAS methods, with results expressed on a dry weight basis. Based on the one-way ANOVA, the F_calculated_ of each element is also shown in comparison with the set F_critical_. Overall, every element varied significantly between samples, since all F_calculated_ values (9.96–1252.51) were higher than the F_critical_ value (1.75) (Table S2).

The mean/median content of essential trace elements in the hemp samples followed the decreasing order: Zn (61.16/57.54 mg/Kg) > Fe (16.60/8.37 mg/Kg) > Cu (6.21/2.28 mg/Kg) > Se (0.32/0.12 mg/Kg) > Mo (0.20/0.11 mg/Kg) > Co (0.13/0.01 mg/Kg) > Cr (0.11/0.03 mg/Kg). However, the great dissimilarity between the mean and median values of every metal reflects a consistent value dispersion over the investigated sample range. Overall, the highest F values were highlighted for Zn (461.89) Fe (421.38), Se (211.54) and Co (120.72) thus, indicating that these essential elements were responsible for the highest variation within the samples (Table S2). 

Considering the producing areas, Sicilian hemps showed the highest levels of Fe (32.73–49.08 mg/Kg), Zn (90.54–150.43 mg/Kg) and Mo (0.48–0.76 mg/Kg), while inflorescences from Lazio had the lowest levels of Fe (1.36–4.49 mg/Kg) and Mo (0.02–0.06 mg/Kg). The lowest levels of Zn were revealed in samples from Lombardy (6.30–18.07 mg/Kg).

However, hemps from Lombardy were characterized by the greatest Cr and Cu contents (18.55–22.80 mg/Kg and 0.30–0.47 mg/Kg); whereas samples from Apulia and Lazio showed the most abundant levels, respectively of Co and Se (0.33–0.93 mg/Kg and 0.88–1.08 mg/Kg). Conversely, samples from Lazio were characterized by the lowest concentrations of Cr (<LOD–0.01 mg/Kg), Cu (0.05–0.35 mg/Kg) and Co (<LOD–0.01 mg/Kg) and hemps from Lombardy with the smallest levels of Se (0.01–0.09 mg/Kg) (Table S2). The variability of each trace essential element between different varieties was less pronounced than that between different production areas, due to more similar concentration values from one variety to another. The Antal variety showed slightly higher levels of Cr (0.00–0.44 mg/Kg), Mo (0.05–0.76 mg/Kg) and Cu (0.22–22.80 mg/Kg) than other varieties. Kompolti hemp was marked by increased levels of Se (0.09–1.07 mg/Kg) and Fe (2.44–44.03 mg/Kg); whereas Finola inflorescences had greater levels of Zn (18-07-110.67 mg/Kg) than other varieties (Table S2). In the investigated hemps, the mean/median content of probably essential trace elements showed the decreasing order: Mn (21.19/17.50 mg/Kg) > Ni (0.45/0.11 mg/Kg). Once again, the difference between mean and median values for both analytes is indicative of a significant value dispersion over the considered samples. Interestingly, the F value calculated for Mn (650.83) was among the highest Fcalculated values of the study (Table S2). 

With respect to the provenience, Sicilian inflorescences showed the highest Mn contents (30.23–72.21 mg/Kg) followed by hemps produced in Apulia (12.04–20.52 mg/Kg) and Lombardy (6.67–35.47 mg/Kg). On the other hand, hemps from Lombardy showed the highest Ni levels (1.07–2.11 mg/Kg) followed by Sicilian samples (0.05–0.15 mg/Kg) (Table S2). Considering the variety factor, Futura 75 and Carmagnola hemps highlighted the highest and lowest levels of Mn (respectively, 16.18–72.21 mg/Kg and 1.67–30.23 mg/Kg); whereas Finola samples showed just slightly higher Ni contents (18-07-110.67 mg/Kg) than other varieties (Table S2). 

The mean/median concentrations of potentially toxic trace elements were revealed in the investigated samples according to the order: Al (25.99/5.91 mg/Kg) > Pb (0.14/0.03 mg/Kg) > Cd (0.009/0.000 mg/Kg) > Hg (0.005/0.000 mg/Kg) > As (0.004/0.000 mg/Kg). The consistent gap between the mean and median content of Al and Pb points out a relevant variation of such metals over hemp samples. Interestingly, Al revealed the highest F value (1252.51) of the study by one-way ANOVA. However, the gap narrows for the mean/median concentrations of Cd, As and Hg, thus, suggesting a lower variability of hemps in relation to these heavy metals. Coherently, these elements showed the lowest F values (9.96–14.99). 

With reference to the geographical origin, Al and Pb were greatly accumulated in Apulian hemps (Al: 23.86–134.07 mg/Kg and Pb: 0.31–0.79 mg/Kg) and, to follow, in samples from Sicily (Pb 0.02–0.08 mg/Kg) and Lombardy (Pb: 1.26–38.33 mg/Kg). Among other heavy metals, Cd and Hg were most abundant, respectively, in Sicilian (0.02–0.06 mg/Kg) and Apulian (0.02–0.04 mg/Kg) inflorescences. Most of the potentially toxic trace elements showed comparable contents in the different hemp varieties, except for Al, which resulted at higher levels in the Tiborzallasi (4.13–134.07 mg/Kg) and Kompolti (2.00–112.6 mg/Kg) varieties (Table S2). 

Recent efforts have been devoted to the assessment of inorganic elements in relation to different geographical producing areas and/or hemp varieties [4,10,22]. Among essential and probably essential trace metals, Amendola and colleagues [22], as well as Zafeiraki and coworkers [10] and Douvris et al. [33], highlighted Zn, Fe Cu and Ni as the most abundant elements. However, they were generally found at much higher and non-comparable concentrations with respect to the contents revealed in the present study. Interestingly, investigated hemps showed higher levels of Se than those shown in literature (0.10–0.15 mg/Kg) [10,22]. Ultimately, the profile of inorganic elements of light hemp highly varies depending on several factors related to its cultivation (i.e., variety of the plant, geopedoclimatic context, agronomic practices, soil properties and not least, the proximity to industrial plants or pollution sources) and its subsequent processing (i.e., drying methods and storage conditions) [4]. For example, elements such as Cd, Cr, Cu, Ni, Pb and Zn are highly influenced by the pH value of the soil. Precisely, they become more bioavailable under acidic soil conditions [4].

However, if, on one hand, the accumulation of some inorganic elements may be advantageous (i.e., essential trace elements), on the other hand, it is not, because, as already described in the introduction section, hemp remarkably accumulates also potentially toxic elements, which may cause hazardous effects to the consumer. In fact, heavy metals fall into the US Federal Drug Administration Class 1 category substances, i.e., “human toxicants that have limited or no use in the manufacture of pharmaceuticals”. Elements such as As, Cd and Ni are classified by the International Agency for Research on Cancer as carcinogenic to humans (Group 1), and their inhalation may also contribute to cardiovascular disease and smoking-related lung diseases. 

The EU has not yet established a regulatory framework concerning the monitoring of hazardous toxicants in cannabis products, including inorganic elements. Nonetheless, data from this study can be compared with the limits set by the WHO framework recommending the continuous monitoring of raw herbal materials intended for medicinal use in terms of potentially toxic metals, other than pesticide residues [34,35], as well as with the thresholds fixed by the US Pharmacopeia (USP) based on a typical consumption of 10 g/day of cannabis products for medical purposes [36].

With reference to the WHO framework, it was found that the experimental concentrations of Cr and heavy metals were lower than fixed limits in all hemps. Differently from WHO, the USP regulates a larger number of essential (i.e., Mo, Cu, Co and Cr) and probably essential metals (i.e., Ni), as well as potentially toxic elements (i.e., Pb, As, Cd and Hg), generally established for the latter lower thresholds than WHO limits. As shown in Table 3, all Italian hemps may be safely ingested due to sub-threshold contents of Mo, Cu, Co, Cr, As, Cd and Hg. However, among investigated samples, two Apulian inflorescences from Finola and Tiborzallasi varieties exceeded the safety ingestion limits for Pb. A different situation was observed for the consumption of Italian hemps via inhalation. In fact, all Italian hemps may be safely inhaled due to sub-limit levels of Mo, As, Cd and Hg. Concerning the other regulated elements, all hemp varieties from Lombardy showed levels of Cu, Cr and Ni above the fixed limits, while all investigated varieties of Apulian cannabis were above the threshold set for Co. Additionally, Apulian products from the varieties Finola and Tiborzallasi accumulated levels of Pb hazardous to inhalation. Overall, the discussed results pointed out that Apulia and Lombardy represented the most contaminated areas in terms of potentially toxic elements and that hemp varieties such as Finola and Tiborzallasi were more susceptible to the accumulation of toxic heavy metals. 

Cases of metal toxicity from cannabis consumption are described in literature. Busse and coworkers [37] highlighted that regular consumers of cannabis in Leipzig reported typical symptoms of Pb intoxication (i.e., abdominal cramps, nausea, anemia, fatigue and in most cases neurologic symptoms) due to marijuana adulterated with Pb during processing. Combemale and colleagues [38] asserted a case of arteritis associated to the consumption of cannabis with very high levels of As. Overall, the combustion and deep inhalation of cannabis products may expose the consumers to very high levels of metals and make them more susceptible to toxic metals, thus, creating a variety of adverse health effects [39,40]. 

### 3.3. PCA of Mineral Elements and Cannabinoids

A PCA was carried out by considering a data matrix with 17 columns (i.e., Al, Cr, Mn, Fe, Co, Ni, Cu, Zn, As, Se, Mo, Cd, Pb, Hg, THC, CBD and CBN) and 24 rows (i.e., number of representative hemp samples) in order to visually reduce data dimensionality and identify those combinations of variables most responsible for sample variability, namely the principal components (PCs).

**Figure 5:**
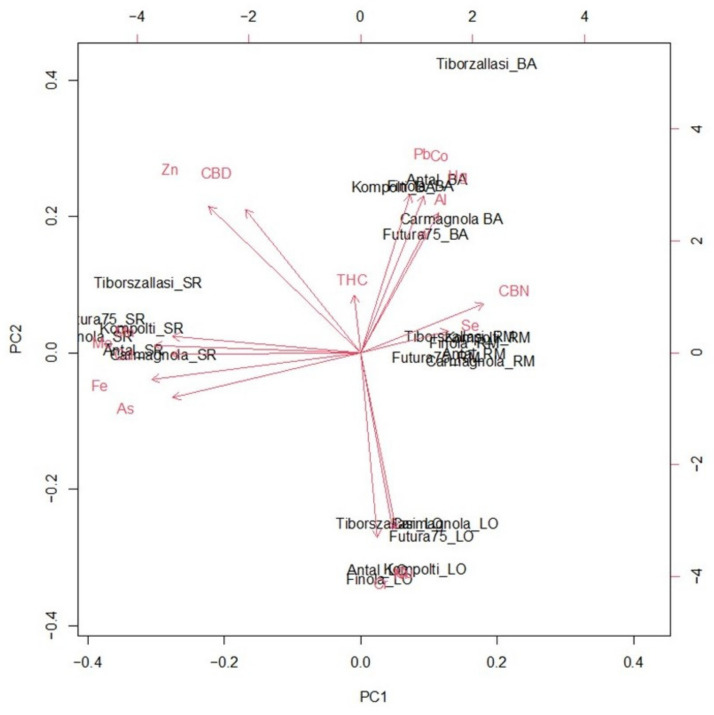
Two-dimensional principal component analysis (PCA) biplot of the first two principal components (PCs) obtained from the variables (i.e., inorganic elements and cannabinoids) investigated in the Italian hemp samples.

Most sample variability was described by the first two PCs, namely PC1 and PC2, which accounted, respectively, for 33.22% and 28.39% of the total variance. The biplot resulting from the first two PCs is reported in Figure 5. 

Most of analyzed samples were separated on PC2 according to the geographical origin rather than botanical variety. Only hemp varieties from Sicily are plotted on PC1 (Figure 5). Specifically, all Apulian hemps were marked by the highest mean levels of Al, Pb and Hg and the lowest concentrations of Ni, Cu and Cr. Conversely, samples from Latium were characterized by a positive correlation with Se and CBN, and a negative correlation with Fe, Mo, Mn, Cd and As. However, these elements are plotted in correspondence to Sicilian hemps, thus, being crucial for their discrimination from other hemps. On the other hand, inflorescences produced in Lombardy were distinguished by a strong positive correlation with Cu, Cr and Ni (Figure 5). 

**Figure 6:**
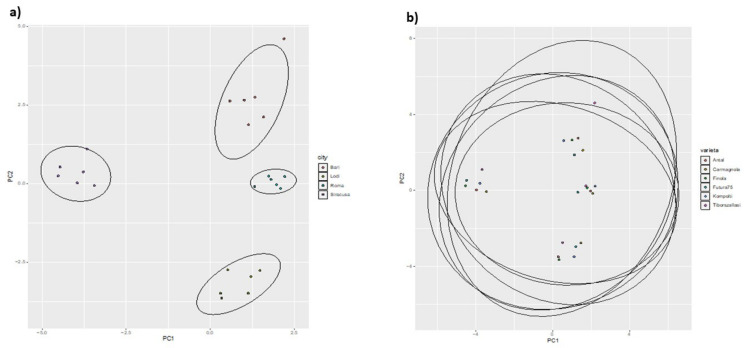
Score plots of the investigated Italian hemps. Drawn ellipses suggest the clustering of samples according to the geographical origin (**a**) botanical variety (**b**).

Accordingly, the score plots (Figure 6) showed that investigated inflorescences sharply clustered according to the production area rather than variety, thus confirming that most variables responsible for the discrimination (i.e., inorganic elements) varied considerably with reference to the geopedoclimatic context in which C. sativa grew.

## 4. Conclusions

Since the introduction of Italian Law 242/2016, the trade of hemp products has literally exploded in small retail shops in many Italian cities. In particular, light cannabis inflorescences are increasingly sold for recreational use, thanks to a lower content of the psychotropic THC, and higher levels of CBD with therapeutic effects. 

In the present study, 72 samples of hemp produced in four Italian regions and belonging to six botanical varieties were screened for trace elements and main cannabinoids. Overall, all samples complied with the Council Directive 2002/53/EC on the common catalogue of varieties of agricultural plant species and, the Italian Law 242/2016 on the provisions for the promotion of hemp cultivation and its agro-industrial supply chain, due to a THC content <0.6%. 

However, the trace element profiles of hemp samples highlighted the urgency to regulate the content of probably essential and potentially toxic elements in hemp products both at the EU and national level, as well as the necessity to constantly monitor such elements to ensure consumers’ safety and health, since several Italian hemps exceeded the current safety limits of Co, Cu, Cr, Ni and Pb set by the available regulatory frameworks (i.e., USP). 

Aside from the assessment of the compliance of Italian hemp with available regulations, experimental data were also statistically interpreted and a PCA pointed out that most investigated variables (i.e., inorganic elements) allowed to discriminate hemp products based on the geographical origin rather than botanical variety, thus, reaching a first milestone toward the development of an effective traceability system for the Italian light cannabis.

## Figures and Tables

**Table 1:** List of the inflorescence samples collected for the present study.

Sample Code	Species	Variety	Geographical Origin	No. Samples
Finola_SR	*C. sativa* L.	Finola	Siracusa (Sicily)	3
Antal_SR		Antal		3
Futura75_SR		Futura75		3
Tiborszallasi_SR		Tiborszallasi		3
Kompolti_SR		Kompolti		3
Carmagnola_SR		Carmagnola		3
Finola_RM		Finola	Rome (Lazio)	3
Antal_RM		Antal		3
Futura75_RM		Futura75		3
Tiborszallasi_RM		Tiborszallasi		3
Kompolti_RM		Kompolti		3
Carmagnola_RM		Carmagnola		3
Finola_BA		Finola	Bari (Apulia)	3
Antal_BA		Antal		3
Futura75_BA		Futura75		3
Tiborszallasi_BA		Tiborszallasi		3
Kompolti_BA		Kompolti		3
Carmagnola_BA		Carmagnola		3
Finola_LO		Finola	Lodi (Lombardy)	3
Antal_LO		Antal		3
Futura75_LO		Futura75		3
Tiborszallasi_LO		Tiborszallasi		3
Kompolti_LO		Kompolti		3
Carmagnola_LO		Carmagnola		3
**Total samples**				**72**

**Table 2:** Analytical validation of ICP-MS and TDA-AAS methods of analysis performed in terms of linearity, limit of detection (LOD), limit of quantification (LOQ), accuracy (*n* = 6) and precision (*n* = 6).

				BCR-402 (White Clover)				
Analyte	R^2^	LOD (mg/Kg)	LOQ (mg/Kg)	Experimental Value (mg/Kg)	Expected Value (mg/Kg)	Recovery (%)	Precision (RSD%)	
							Intraday	Interday
Al *	0.9995	0.067	0.221	1.96	2.00	98.00 ± 0.50	1.1	1.3
Cr	0.9996	0.002	0.007	5.11	5.19	98.46 ± 0.39	0.3	0.5
Mn *	0.9998	0.003	0.010	1.98	2.00	99.00 ± 0.50	1.0	1.4
Fe	0.9995	0.024	0.079	250.1	244.00	102.50 ± 0.45	0.2	0.5
Co	0.9998	0.001	0.003	0.172	0.178	96.63 ± 1.12	0.4	0.7
Ni	0.9996	0.002	0.007	7.99	8.25	96.85 ± 0.56	0.3	0.2
Cu *	0.9992	0.017	0.056	1.92	2.00	96.00 ± 1.00	0.5	0.9
Zn	0.9994	0.061	0.201	24.8	25.2	98.41 ± 0.79	1.2	1.5
As	0.9998	0.001	0.003	0.094	0.093	101.08 ± 1.08	0.9	1.2
Se	0.9993	0.002	0.007	6.65	6.70	99.25 ± 0.15	0.3	0.6
Cd *	0.9999	0.001	0.003	2.00	2.00	100.00 ± 0.50	1.3	1.7
Pb *	0.9997	0.002	0.007	1.98	2.00	99.00 ± 0.50	0.7	0.8
Mo	0.9997	0.002	0.007	6.78	6.93	97.84 ± 0.44	0.3	0.4
Hg *	0.9998	0.002	0.007	1.96	2.00	98.00 ± 1.00	0.6	1.0

* Analyte not present in the certified matrix.

**Table 3:** Number of Italian hemps exceeding the World Health Organization (WHO) and US Pharmacopeia (USP) limits in terms of inorganic elements. WHO limits refer to the maximum content of elements allowable in raw herbal materials intended for medicinal use. USP sets the threshold of elements for both oral intake and inhalation of cannabis products, considering the consumption of 10 g/day.

Element	WHO 2007		Oral Concentration		Inhalation Concentration	
	Limit (mg/Kg)	No. Samples	Limit (mg/Kg)	No. Samples	Limit (mg/Kg)	No. Samples
Mo	-	-	300	0	1	0
Cu	-	-	300	0	3	8
Co	-	-	5	0	0.3	6
Cr	2	0	1100	0	0.3	6
Ni	-	-	20	0	0.5	6
Pb	10	0	0.5	2	0.5	2
As	5	0	1.5	0	0.2	0
Cd	0.3	0	0.5	0	0.2	0
Hg	0.2	0	3	0	0.1	0

## Data Availability

Not applicable.

## Supplementary Materials

The following supporting information can be downloaded at: https://www.mdpi.com/article/10.3390/toxics10120758/s1, Table S1. Percentage content of major cannabinoids (i.e., THC, CBD and CBN) in the different samples of inflorescences from *C. sativa* L. Results are expressed as mean ± standard deviation (mg/Kg, dw) of n=3 samples, where every sample was analyzed three times; Table S2. Profile of inorganic elements of the different samples of inflorescences from *C. sativa* L. Results are expressed as mean ± standard deviation (mg/Kg, dw) of n=3 samples, where every sample was analyzed three times.
