# Supplementary material for: Monitoring Cannabinoids and the Safety of the Trace Element Profile of Light Cannabis sativa L. from Different Varieties and Geographical Origin
Source: Toxics. 2022 Dec 5;10(12):758. doi: 10.3390/toxics10120758 (PMC9785383; doi:10.3390/toxics10120758)
Supplement: Supplementary file 1 [file toxics-10-00758-s001.zip › toxics-2026002-supplementary.pdf]

Supplementary material

Table S1. Percentage content of major cannabinoids (i.e., THC, CBD and CBN) in the different samples of inflorescences from *C. sativa* L. Results are expressed as mean ± standard deviation (mg/Kg, dw) of n=3 samples, where every sample was analyzed three times.

Sample	THC%	CBD%	CBN%
**Finola_SR**	0.28±0.03	5.55±0.99	0.07±0.00
**Antal_SR**	0.19±0.04	4.86±0.10	0.08±0.02
**Futura75_SR**	0.33±0.03	7.65±0.09	0.12±0.05
**Tiborszallasi_SR**	0.37±0.06	7.76±0.09	0.11±0.00
**Kompolti_SR**	0.34±0.06	7.76±0.13	0.07±0.00
**Carmagnola_SR**	0.08±0.03	5.30±1.06	0.04±0.00
**Finola_RM**	0.42±0.05	1.05±0.05	0.14±0.03
**Antal_RM**	0.37±0.12	1.24±0.08	0.22±0.00
**Futura75_RM**	0.33±0.06	2.12±0.12	0.11±0.01
**Tiborszallasi_RM**	0.36±0.06	3.26±1.21	0.20±0.06
**Kompolti_RM**	0.22±0.03	1.90±0.06	0.26±0.08
**Carmagnola_RM**	0.15±0.03	2.52±0.19	0.27±0.07
**Finola_BA**	0.25±0.04	5.74±0.05	0.12±0.01
**Antal_BA**	0.25±0.06	7.62±0.09	0.26±0.01
**Futura75_BA**	0.11±0.05	6.35±0.62	0.16±0.01
**Tiborszallasi_BA**	0.39±0.06	6.03±0.94	0.30±0.05
**Kompolti_BA**	0.13±0.04	8.78±2.03	0.05±0.01
**Carmagnola_BA**	0.20±0.07	5.45±0.97	0.14±0.01
**Finola_LO**	0.18±0.05	2.92±0.06	0.03±0.01
**Antal_LO**	0.27±0.04	2.35±0.49	0.20±0.02
**Futura75_LO**	0.30±0.05	1.72±0.06	0.24±0.05
**Tiborszallasi_LO**	0.10±0.05	3.03±0.11	0.20±0.06
**Kompolti_LO**	0.13±0.04	1.74±0.06	0.07±0.00
**Carmagnola_LO**	0.15±0.05	2.57±0.07	0.07±0.00
**Mean**	0.24	4.39	0.14
**Median**	0.25	4.06	0.13
**Standard deviation**	0.10	2.44	0.08
**Fcalculated**	11.35	45.30	18.01

*Fcrit =1.75 at α<0.001.

Table S2. Profile of inorganic elements of the different samples of inflorescences from *C. sativa* L. Results are expressed as mean ± standard deviation (mg/Kg, dw) of n=3 samples, where every sample was analyzed three times.

Sample	As	Cd	Co	Cr	Cu	Fe	Mn	Mo	Ni	Pb	Se	Zn	Al	Hg
**Finola_SR**	0.01±0.00	0.06±0.03	0.01±0.00	0.03±0.01	2.61±0.11	47.56±2.08	49.96±0.15	0.50±0.05	0.15±0.02	0.04±0.02	0.17±0.02	110.67±9.42	4.59±0.11	<LOD
**Antal_SR**	0.01±0.01	0.02±0.01	<LOD	0.05±0.02	2.73±0.11	49.08±0.14	39.60±2.08	0.76±0.10	0.12±0.04	0.08±0.03	0.12±0.04	111.24±3.64	5.01±0.07	<LOD
**Futura75_SR**	0.01±0.01	0.04±0.02	<LOD	0.11±0.02	0.67±0.13	38.56±2.36	72.21±0.19	0.48±0.03	0.10±0.04	0.06±0.03	0.15±0.04	103.81±0.96	1.88±0.07	<LOD
**Tiborszallasi_SR**	0.01±0.00	0.02±0.01	0.01±0.00	0.04±0.01	0.89±0.07	40.81±3.93	41.16±1.24	0.54±0.09	0.05±0.02	0.02±0.01	0.11±0.03	150.43±8.07	8.78±0.24	<LOD
**Kompolti_SR**	0.01±0.01	0.02±0.02	<LOD	0.06±0.02	3.06±0.11	44.03±2.74	36.18±0.87	0.66±0.08	0.11±0.04	0.05±0.02	0.14±0.02	97.36±1.41	6.81±0.19	<LOD
**Carmagnola_SR**	0.01±0.01	0.04±0.02	0.01±0.01	<LOD	0.56±0.12	32.73±2.90	30.23±1.27	0.54±0.09	0.12±0.06	0.07±0.02	0.11±0.04	90.54±2.06	1.22±0.20	<LOD
**Finola_RM**	<LOD	<LOD	<LOD	0.01±0.01	0.33±0.08	4.49±0.47	3.59±0.32	0.06±0.02	0.02±0.01	0.01±0.00	0.97±0.07	45.81±0.57	4.50±0.33	0.01±0.00
**Antal_RM**	<LOD	<LOD	<LOD	<LOD	0.22±0.11	2.99±0.16	2.15±0.15	0.05±0.02	0.02±0.01	0.02±0.01	0.93±0.09	43.87±0.80	2.06±0.17	<LOD
**Futura75_RM**	<LOD	<LOD	0.01±0.00	<LOD	0.05±0.02	1.87±0.30	18.44±0.34	0.03±0.01	0.01±0.00	<LOD	1.07±0.03	44.22±0.45	1.27±0.17	<LOD
**Tiborszallasi_RM**	<LOD	<LOD	<LOD	0.01±0.00	0.09±0.03	3.57±0.39	2.04±0.16	0.03±0.02	0.01±0.00	0.01±0.01	1.08±0.06	52.39±0.83	4.13±0.16	<LOD
**Kompolti_RM**	<LOD	<LOD	<LOD	<LOD	0.35±0.06	2.44±0.22	2.58±0.31	0.02±0.01	0.03±0.02	<LOD	1.07±0.06	54.43±0.46	2.00±0.08	0.01±0.00
**Carmagnola_RM**	<LOD	<LOD	<LOD	0.01±0.00	0.10±0.05	1.36±0.31	1.67±0.27	0.04±0.01	0.01±0.00	0.01±0.00	0.88±0.11	39.94±1.51	1.15±0.17	<LOD
**Finola_BA**	<LOD	<LOD	0.46±0.10	0.02±0.01	2.27±0.22	5.38±0.71	16.18±3.28	0.12±0.07	0.07±0.03	0.79±0.12	0.13±0.03	84.42±1.95	23.86±0.29	0.02±0.02
**Antal_BA**	<LOD	<LOD	0.35±0.07	0.03±0.01	3.09±0.10	4.17±0.32	17.67±1.24	0.13±0.06	0.11±0.04	0.44±0.08	0.15±0.05	80.54±2.35	25.04±2.87	0.03±0.02
**Futura75_BA**	<LOD	<LOD	0.39±0.08	0.01±0.01	1.28±0.20	6.44±0.27	16.18±1.61	0.01±0.00	0.02±0.01	0.41±0.11	0.11±0.04	67.60±1.56	87.29±2.40	<LOD
**Tiborszallasi_BA**	<LOD	<LOD	0.93±0.09	<LOD	2.82±0.18	8.86±1.90	20.52±1.03	0.13±0.03	0.19±0.11	0.61±0.15	0.11±0.06	72.93±1.90	134.07±7.15	0.04±0.02
**Kompolti_BA**	<LOD	<LOD	0.75±0.10	0.02±0.01	2.31±0.17	7.88±0.21	19.57±0.35	0.03±0.02	0.15±0.05	0.35±0.09	0.15±0.02	85.40±1.79	122.60±3.02	<LOD
**Carmagnola_BA**	<LOD	<LOD	0.33±0.05	<LOD	1.24±0.11	3.54±0.22	12.04±1.14	0.01±0.01	0.01±0.01	0.31±0.11	0.10±0.05	60.66±2.33	102.08±2.77	0.02±0.01
**Finola_LO**	<LOD	0.01±0.00	0.01±0.00	0.47±0.07	20.89±3.22	21.13±0.45	15.98±0.13	0.21±0.09	2.11±0.05	0.03±0.01	0.03±0.02	18.07±2.44	15.44±0.21	<LOD
**Antal_LO**	0.01±0.01	<LOD	0.01±0.01	0.44±0.10	22.80±3.64	22.57±0.12	17.34±0.32	0.18±0.05	1.88±0.16	0.03±0.01	0.01±0.01	12.80±1.78	21.72±0.26	<LOD
**Futura75_LO**	<LOD	0.01±0.00	0.02±0.01	0.33±0.04	19.62±5.10	9.28±0.42	35.47±2.60	0.01±0.01	1.92±0.16	0.01±0.01	0.01±0.00	12.43±3.23	4.72±0.27	<LOD
**Tiborszallasi_LO**	0.01±0.00	<LOD	0.01±0.01	0.36±0.06	20.97±3.38	23.39±0.25	17.91±0.30	0.10±0.05	1.09±0.03	0.03±0.01	0.05±0.03	13.47±1.63	38.33±3.55	<LOD
**Kompolti_LO**	<LOD	<LOD	<LOD	0.39±0.07	21.43±3.86	14.63±0.25	6.67±0.20	0.13±0.05	1.55±0.32	0.02±0.02	0.09±0.07	6.30±2.27	14.17±0.18	<LOD
**Carmagnola_LO**	<LOD	<LOD	<LOD	0.30±0.05	18.55±3.68	1.64±1.41	13.21±0.49	0.01±0.00	1.07±0.06	<LOD	0.01±0.00	8.54±2.17	1.26±0.28	<LOD
**Mean**	**0.004**	**0.009**	**0.13**	**0.11**	**6.21**	**16.60**	**21.19**	**0.20**	**0.45**	**0.14**	**0.32**	**61.16**	**25.99**	**0.005**
**Median**	**0.000**	**0.000**	**0.01**	**0.03**	**2.29**	**8.37**	**17.50**	**0.11**	**0.11**	**0.03**	**0.12**	**57.54**	**5.91**	**0.000**
**Standard deviation**	**0.006**	**0.017**	**0.26**	**0.16**	**8.64**	**16.58**	**17.36**	**0.24**	**0.71**	**0.22**	**0.40**	**38.80**	**39.72**	**0.011**
**Fcalculated**	**14.99**	**9.96**	**120.72**	**66.16**	**59.92**	**421.38**	**650.83**	**66.53**	**68.87**	**44.91**	**211.54**	**461.89**	**1252.51**	**14.59**

*Fcritical =1.75 at α<0.001
